# Achievement of individualized treatment targets in patients with comorbid type-2 diabetes and hypertension: 6 months results of the DIALOGUE registry

**DOI:** 10.1186/s12902-015-0020-7

**Published:** 2015-05-02

**Authors:** Roland E Schmieder, Anselm K Gitt, Cornelia Koch, Peter Bramlage, Taoufik Ouarrak, Diethelm Tschöpe, For the DIALOGUE study group

**Affiliations:** Medizinische Klinik 4, Nephrologie und Hypertensiologie, Universitätsklinikum Erlangen, Ulmenweg 18, 91054 Erlangen, Germany; Stiftung Institut für Herzinfarktforschung, Ludwigshafen, Germany; Medizinische Klinik B, Herzzentrum Ludwigshafen, Ludwigshafen, Germany; Novartis Pharma GmbH, Nürnberg, Germany; Institut für Pharmakologie und Präventive Medizin, Mahlow, Germany; Herz- und Diabeteszentrum Nordrhein-Westfalen, Bad Oeynhausen, Germany; Stiftung “Der herzkranke Diabetiker” in der Deutschen Diabetes-Stiftung, Georgstrasse 11, 32545 Bad Oeynhausen, Germany

## Abstract

**Background:**

Patients with type-2 diabetes mellitus (T2DM) and hypertension have increased risk of cardiovascular disease (CVD). We studied individualized treatment targets and their achievement in clinical practice.

**Methods:**

DIALOGUE is a prospective, multi-center registry in patients with both T2DM and hypertension.

**Results:**

Patients (n = 6,586) had a baseline fasting glucose (8.5 ± 2.8 mmol/l), postprandial glucose (10.9 ± 3.4 mmol/l), and HbA1c (7.8 ± 2.1%) levels indicated poor glycemic control. Baseline systolic and diastolic BP were 140.3 ± 15.7 and 82.6 ± 9.5, respectively. Patients were categorized by HbA1c treatment goals: ≤6.5% (strict), >6.5 to ≤7.0% (medium), and >7.0 to ≤7.5% (loose). When considering systolic BP (SBP) targets (≤130 mmHg [strict], >130 to ≤135 mmHg [medium], and >135 to ≤140 mmHg [loose]), patients with strict SBP treatment goals displayed similar characteristics to those with strict HbA1c targets. Although approximately 70% of patients received both strict HbA1c and SBP targeting, overall treatment goals remained unmet in all HbA1c target groups at the 6-month follow-up. SBP targets were not reached in the strict and medium groups, but were achieved in the loose treatment group. Specific predictors for choosing loose SBP or HbA1c treatment goals were identified, including SBP/HbA1c levels and various comorbidities.

**Conclusions:**

Individualized glucose and BP targets were selected by treating physicians based on patient characteristics and overall comorbidity. While treatment goals were not consistently met using various antidiabetic and antihypertensive therapies, our analyses indicated that the strictly targeted patient populations maintained lower overall HbA1c and SBP levels at 6 months.

## Background

A large body of evidence indicates that type-2 diabetes mellitus (T2DM) is an important independent risk factor for cardiovascular disease (CVD). In fact, those with diabetes are 2 to 4 times more likely to develop CVD, which is the leading cause of mortality in patients with T2DM [1]. In addition, the prevalence of hypertension is more than double in diabetic patients compared to those with normal blood glucose levels [1], making it the most common comorbid disease associated with T2DM [2]. Hypertension has been found to increase the risk of nephropathy, retinopathy, left ventricular hypertrophy, and cardiovascular events in patients with T2DM [1].

Current guidelines have recommended a multi-factorial approach for treating diabetic patients with hypertension, involving simultaneous targeting of blood pressure (BP) and glucose levels [3]. Although there has been a recent focus on individualized treatment targets in patients with T2DM [4], guidelines have not been sufficiently translated into clinical practice [5]. Moreover, adequate guidance with regard to individualized BP targets remains to be established [6,7]. Thus, in order to define effective criteria for individualized treatment approaches, information regarding patient characteristics that might be associated with specific HbA1c and BP target groups as well as investigation into the efficacy of meeting individualized therapeutic goals set by physicians in daily clinical practice are fundamental. This is especially important considering that drugs that perform well within specialized populations in clinical trials are often less effective when employed in clinical practice. Thus, it is also essential to carefully evaluate whether treatment targets can be met using specific antidiabetic and antihypertensive therapies in unselected patient populations and within real clinical settings. For this reason, comparisons between different drug classes are required.

The ongoing DIALOGUE registry represents the first study to assess the effectiveness, tolerability, and impact of different therapeutic approaches in patients with T2DM and hypertension while applying newly established individualized treatment targets recommended by the American Diabetes Association (ADA) and the European Association for the Study of Diabetes (EASD) [4]. Here, we aimed to characterize patients based on their chosen therapeutic targets as well as actual target achievement rates (overall and by comorbidity). Furthermore, we identified patient characteristics associated with loose treatment goals.

## Methods

### Study design

DIALOGUE is an ongoing, prospective, observational, non‐interventional, multi-center disease registry with a follow-up of up to 24 months (i.e., 6, 12 and 24 months) in Germany. Diabetologists and primary care physicians are in charge of continued patient enrollment at selected centers, which were chosen from a database (Stiftung Institut für Herzinfarktforschung) to be representative of ambulatory care for diabetes and hypertension. This registry is being conducted in accordance with the Declaration of Helsinki and adheres to the principles of Good Epidemiology Practice. Moreover this investigation has followed applicable regulatory requirements, and the study protocol was approved by the ethics committee of the Ruhr University (Bochum, Germany). In addition, all patients provided written informed consent, and DIALOGUE was registered in the database of the Verband forschender Arzneimittelhersteller (http://www.vfa.de/de/arzneimittel-forschung/datenbanken-zu-arzneimitteln/nisdb). The study protocol, as well as primary and secondary objectives of DIALOGUE, have been previously published in detail [8]. Decisions regarding individual therapies and treatment goals (HbA1c and BP) were made solely by the attending physician based on their clinical assessment.

### Patients

Patients were consecutively enrolled based on the following criteria: at least 18 years old; T2DM and manifested comorbid hypertension; current use of oral mono‐ or dual combination antidiabetic therapy; treating physician considered blood glucose lowering medication as inadequate and/or not safe/tolerable; the physician added an additional oral drug or switched drug treatment to achieve glycemic control (excluding glucagon-like peptide [GLP-1] analogues and insulin). Patients were not eligible for inclusion if any of the following criteria applied: current participation in a randomized controlled trial; not under regular supervision of the treating physician during the study; use of GLP-1 analogues or insulin before enrollment; treated with aliskiren in a dual renin-angiotensin-aldosterone system (RAAS) blockade; pregnancy; diabetes secondary to malnutrition, infection or surgery; maturity onset diabetes of the young; and known cancer.

### Data collection and quality assurance

Data were recorded using a web-based electronic case report form (eCRF). Among other information, the following information was collected: patient characteristics (basic characteristics, medical history, and comorbidities); medical therapy for secondary prevention of cardio‐vascular complications; glucose profile (fasting glucose, post‐prandial glucose, HbA1c); BP; and body mass index (BMI). Office BP was assessed with standard oscillometric devices available at the physician’s office with a calibration validation. Data quality was ensured upon eCRF entry, prior to creation of the analysis data set, and through on-site monitoring (2% of the sites randomly selected).

### Statistical analyses

Continuous variables were summarized using standard statistics (i.e., mean, standard deviation, minimum, median, maximum, lower and upper quartile), whereas percentages were calculated for categorical data. Comparisons between treatment groups were performed using Pearson´s chi-squared test for categorical variables and the Kruskal–Wallis test for continuous measures. Predictors for target group selection were identified through multivariate analysis. All statistical analyses were performed using SAS (release 9.2 or higher; Cary, NC, USA). P-values ≤ 0.05 were considered to be significant.

## Results

Recruitment for DIALOGUE began in July 2012, with 8,632 patients documented as of May 2014. Here, we present baseline data for these patients and analyze data from those completing the 6-month follow-up (n = 6,586).

### Baseline patient characteristics

The characteristics of the study participants are presented in Table 1. Patients displayed a median age of 65 years, and less than a quarter were > 75 years old. Approximately half of the patients were female. There was a large proportion of obese patients, and more than one-tenth were recorded as current smokers. Mean diabetes duration was approximately 6 years. Baseline fasting and postprandial glucose measurements, as well as HbA1c levels, indicated poor glycemic control in our cohort (as expected by study design). Finally, mean BP was found to be 140 ± 15.7/83 ± 9.5 (systolic/diastolic), and the average heart rate was within normal range.

**Table 1:** **Baseline patient characteristics for all subjects and each HbA1c target group**

**Baseline characteristic**	All subjects (n = 8,632*)	**HbA1c ≤ 6.5% (n = 3,369)**	**HbA1c > 6.5% to ≤ 7.0% (n = 3,644)**	**HbA1c > 7.0% to ≤ 7.5% (n = 1,618)**	**P-values for the comparison of 3 groups**
Age (years)	65.0 (57.0-74.0)	64.0 (55.0-73.0)	67.0 (59.0-74.0)	66.0 (58.0-75.0)	<0.0001
Age > 75 years (%)	19.7	17.7	20.5	22.0	<0.001
Female sex (%)	45.6	46.2	45.5	44.4	0.47
BMI (kg/m^2^)	31.2 ± 5.9	31.1 ± 5.9	31.3 ± 5.8	31.4 ± 6.0	0.38
BMI > 30 kg/m^2^ (%)	52.0	51.3	52.1	53.1	0.47
Waist circumference (cm)	107.6 ± 14.5	107.0 ± 15.0	108.0 ± 14.0	108.0 ± 14.7	0.50
Current smokers (%)	11.8	11.4	12.0	11.9	0.73
Diabetes duration (months)	67.9 (31.7-117.3)	55.7 (24.8-100.6)	75.1 (35.7-122.4)	82.7 (40.4-131.8)	<0.0001
Fasting glucose (mmol/l)	8.5 ± 2.8	7.8 ± 2.6	8.8 ± 2.6	9.7 ± 3.1	<0.0001
Postprandial glucose (mmol/l)	10.9 ± 3.4	10.0 ± 3.2	11.2 ± 3.3	12.1 ± 3.7	<0.0001
HbA1c (%)	7.8 ± 2.1	7.2 ± 1.3	8.0 ± 2.8	8.6 ± 1.5	<0.0001
≤6.5% (%)	14.7	28.9	7.1	2.0	<0.0001
>6.5% to ≤ 7.0% (%)	17.4	25.6	16.2	2.5	<0.0001
>7.0% to ≤ 7.5% (%)	19.8	17.7	23.1	16.8	<0.0001
SBP (mmHg)	140.3 ± 15.7	139.5 ± 15.9	140.7 ± 15.5	141.3 ± 15.6	<0.0001
DBP (mmHg)	82.6 ± 9.5	82.4 ± 9.8	82.7 ± 9.2	83.0 ± 9.4	<0.01
HR (beats/min)	75.0 ± 10.0	74.7 ± 10.4	75.0 ± 9.4	75.9 ± 10.5	<0.0001

Legend: HbA1c, glycated hemoglobin; body mass index, BMI; SBP, systolic blood pressure; DBP, diastolic blood pressure; HR, heart rate; *for one patient no HbA1c target was indicated; data are provided as medians (interquartile range), percent or mean ± standard deviation.

### Individualized treatment targets

**Figure 1:**
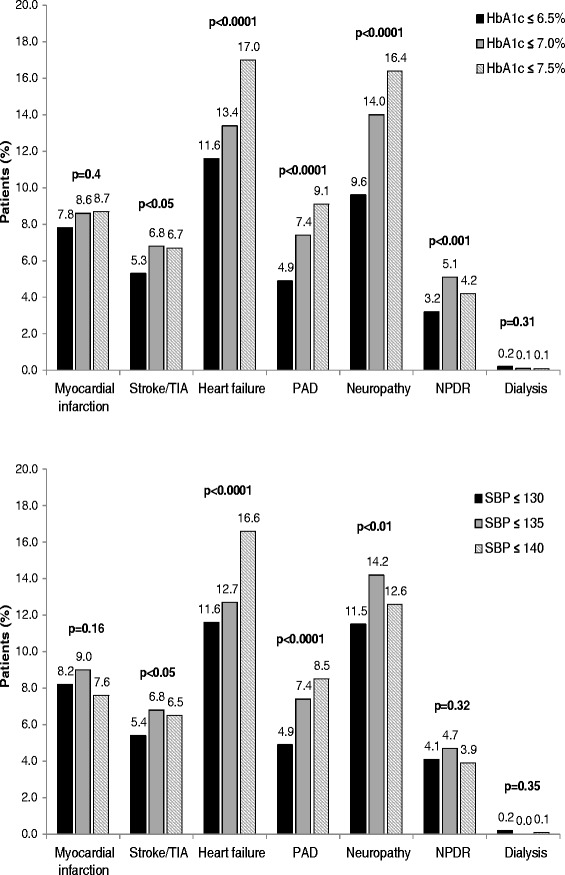
Comorbidities according to HbA1c (upper panel) and BP (lower panel) target groups. Legend: HbA1c, glycated hemoglobin; SBP, systolic blood pressure; TIA, transient ischemic attack; PAD, peripheral arterial disease; NPDR, non-proliferative diabetic retinopathy.

Patients were categorized into three groups based on initial HbA1c treatment goals: ≤6.5% (strict group), >6.5 to ≤7.0% (medium), and >7.0 to ≤7.5% (loose) (Table 1). The loose treatment group contained approximately half as many patients as the other two groups. Patients in the strict target group were younger and displayed a shorter disease duration compared to the other treatment groups (p < 0.0001). In addition, they presented lower fasting/postprandial blood glucose and HbA1c values (all p < 0.0001). Moreover, patients with strict HbA1c goals displayed significantly lower BP and heart rate as well as significantly less comorbid disease at baseline than the other groups (Figure 1, upper panel).

Similarly, patients were also divided based on target systolic BP (SBP: ≤130 mmHg [strict], >130 to ≤135 mmHg [medium], and >135 to ≤140 mmHg [loose]) (Table 2). When considering patients within the SBP target groups, we found that those with strict treatment goals were significantly younger and had a shorter diabetes duration (p < 0.0001). Also, they displayed better glycemic control (p < 0.0001), lower BP measurements (p < 0.0001), and significantly less co-morbid disease when compared to the other SBP target groups (Figure 1, lower panel).

**Table 2:** **Baseline patient characteristics by SBP target group**

**Baseline characteristic**	All subjects (n = 8,632*)	**SBP ≤ 130 mmHg (n = 3,342)**	**SBP > 130 to ≤ 135 mmHg (n = 2,870)**	**SBP > 135 to ≤ 140 mmHg (n = 2,384)**	**P-values for the comparison of 3 groups**
Age (years)	65.0 (57.0-74.0)	64.0 (55.0-72.0)	66.0 (58.0-74.0)	68.0 (59.0-75.0)	<0.0001
Age > 75 years (%)	19.7	16.7	20.5	22.8	<0.0001
Female sex (%)	45.6	46.1	44.8	45.5	0.59
BMI (kg/m^2^)	31.2 ± 5.9	31.1 ± 5.8	31.2 ± 5.7	31.4 ± 6.1	0.48
BMI > 30 kg/m^2^ (%)	52.0	51.4	51.8	52.9	0.53
Waist circumference (cm)	107.6 ± 14.5	107.8 ± 15.1	107.4 ± 13.7	107.5 ± 14.8	0.68
Current smokers (%)	11.8	12.1	11.5	11.6	0.77
Diabetes duration (months)	67.9 (31.7-117.3)	61.4 (27.6-109.7)	71.2 (34.1-120.2)	72.7 (34.1-121.1)	<0.0001
Fasting glucose (mmol/l)	8.5 ± 2.8	8.2 ± 2.7	8.7 ± 2.8	8.8 ± 2.9	<0.0001
Postprandial glucose (mmol/l)	10.9 ± 3.4	10.5 ± 3.2	11.2 ± 3.4	11.1 ± 3.6	<0.0001
HbA1c (%)	7.8 ± 2.1	7.5 ± 1.4	7.9 ± 1.4	8.1 ± 3.4	<0.0001
SBP (mmHg)	140.3 ± 15.7	136.4 ± 15.5	140.7 ± 14.8	145.2 ± 15.4	<0.0001
≤ 130 mmHg (%)	32.0	45.2	28.1	17.8	<0.0001
> 130 to ≤ 135 mmHg (%)	9.2	8.8	12.4	5.8	<0.0001
> 135 to ≤ 140 mmHg (%)	21.7	18.2	22.4	26.1	<0.0001
DBP (mmHg)	82.6 ± 9.5	81.3 ± 9.5	82.9 ± 9.1	84.1 ± 9.7	<0.0001
HR (beats/min)	75.0 ± 10.0	74.5 ± 10.0	75.2 ± 10.2	75.6 ± 10.2	<0.0001

Legend: SBP, systolic blood pressure; BMI, body mass index; HbA1c, glycated hemoglobin; DBP, diastolic blood pressure; HR, heart rate; *for 36 patients no SBP target was indicated; data are provided as medians (interquartile range), percent or mean ± standard deviation.

**Figure 2:**
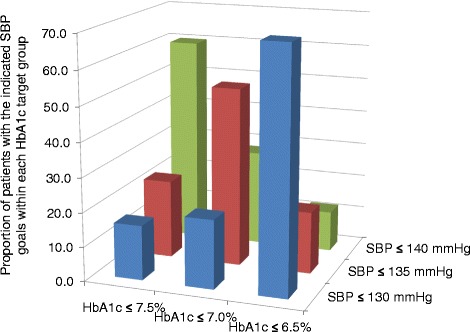
Proportion of patients with the indicated SBP goals within each HbA1c target group. Legend: SBP, systolic blood pressure; HbA1c, glycated hemoglobin. *P < 0.0001 for all comparisons between HbA1c target groups.

Therefore, our data indicated that key similarities existed between patients within the strict HbA1c and SBP target groups. Thus, we further analyzed the relationship between patients with distinct HbA1c and SBP treatment goals. Strikingly, we found that approximately 70% of the patients who received strict HbA1c targeting also had strict SBP treatment goals (Figure 2). Moreover, cross comparison of patients within the medium and loose treatment groups revealed similar results, with 52% and 60% of patients in the respective HbA1c target groups fitting within the corresponding SBP treatment groups.

In particular, factors contributing to the assignment of patients into the loose target groups for SBP and HbA1c were of interest. With this respect, multivariate analysis revealed several adjusted predictors for selecting loose treatment goals (Table 3). We found that loose SBP targets were weakly correlated with age, fasting blood glucose (FBG), heart failure, peripheral artery disease (PAD), and absence of non-proliferative diabetic retinopathy (NPDR); SBP at baseline was the strongest predictor. On the other hand, loose HbA1c targets could be weakly correlated with longer diabetes duration, heart failure, PAD and neuropathy with HbA1c being the strongest predictor.

**Table 3:** **Multivariable adjusted predictors for choosing a loose treatment target**

	**SBP > 135 to ≤ 140 mmHg**		**HbA1c >7.0% to ≤ 7.5%**	
	**uni (OR; 95%CI)**	**multi (OR; 95%CI)**	**uni (OR; 95%CI)**	**multi (OR; 95%CI)**
Age (years) (≥ vs. < median)	*1.37 (1.24-1.51)*	*1.28 (1.04-1.56)*	*1.11 (1.00-1.24)*	0.95 (0.74-1.21)
Female sex (%)	1.00 (0.91-1.10)	1.01 (0.84-1.22)	0.94 (0.85-1.05)	1.22 (0.97-1.54)
BMI (kg/m^2^) (≥ vs. < median)	1.06 (0.96-1.16)	1.07 (0.86-1.34)	1.09 (0.98-1.22)	0.94 (0.72-1.23)
Waist circumference (cm) (≥ vs. < median)	1.08 (0.91-1.27)	1.01 (0.81-1.26)	1.05 (0.87-1.28)	1.00 (0.77-1.31)
Current smokers (%)	0.98 (0.84-1.14)	1.13 (0.85-1.50)	1.02 (0.86-1.21)	1.15 (0.82-1.61)
Diabetes duration (months) (≥ vs. < median)	*1.14 (1.03-1.26)*	0.97 (0.80-1.17)	*1.50 (1.34-1.67)*	*1.36 (1.09-1.70)*
FBG (mmol/l) (≥ vs. < median)	*1.38 (1.24-1.53)*	*1.29 (1.06-1.58)*	*2.31 (2.03-2.62)*	1.24 (0.96-1.59)
HbA1c (%)(≥ vs. < median)	*1.33 (1.20-1.47)*	1.14 (0.93-1.41)	*4.03 (3.58-4.53)*	*3.20 (2.47-4.05)*
SBP (mmHg) (≥ vs. < median)	*2.83 (2.54-3.14)*	*2.23 (1.84-2.70)*	1.25 (1.12-1.40)	1.22 (0.98-1.53)
Prior MI (yes vs. no)	0.87 (0.72-1.04)	0.86 (0.61-1.20)	1.05 (0.86-1.28)	0.85 (0.57-1.27)
Prior stroke/TIA (yes vs. no)	1.09 (0.90-1.33)	0.77 (0.54-1.10)	1.11 (0.89-1.39)	0.64 (0.41-0.99)
Heart failure (yes vs. no)	*1.45 (1.27-1.66)*	*1.71 (1.33-2.21)*	*1.44 (1.23-1.67)*	*1.54 (1.13-2.09)*
PAD (yes vs. no)	*1.43 (1.20-1.72)*	*1.46 (1.02-2.08)*	*1.51 (1.24-1.85)*	*1.92 (1.30-2.85)*
Neuropathy (yes vs. no)	0.99 (0.86-1.15)	0.80 (0.61-1.04)	*1.45 (1.24-1.69)*	*1.60 (1.21-2.12)*
NPDR (yes vs. no)	0.89 (0.70-1.14)	*0.64 (0.41-0.99)*	0.99 (0.75-1.31)	0.83 (0.52-1.31)

Legend: BMI, body mass index; FPG, fasting blood glucose; PPBG, postprandial blood glucose; HbA1c, glycated hemoglobin; SBP, systolic blood pressure; DBP, diastolic blood pressure; HR, heart rate; MI, myocardial infarction; TIA, transient ischemic attack; PAD, peripheral arterial disease; NPDR, non-proliferative diabetic retinopathy.

### Antidiabetic and antihypertensive therapies by target group

We also analyzed therapeutic patterns in the HbA1c and SBP target groups, observing significant differences with regard to specific antidiabetic and antihypertensive therapies (Table 4). The lowest use of various antidiabetic therapies occurred in the strict HbA1c target group. In addition, this group was administered the lowest drug doses (data not shown). In contrast, the medium HbA1c target group showed significantly higher amounts of use of metformin, alpha-glucosidase, and dipeptidyl peptidase-4 (DDP-4) inhibitors, whereas those patients with loose HbA1c targeting were more often administered sulfonylureas and insulin (short-acting, long-acting, and mixed) (p < 0.0001). With regard to antihypertensive therapies, there were fewer significant differences observed between the distinct SBP target groups. However, we found that the loose target group was more often prescribed ACE inhibitors, calcium blockers, diuretics, and other drugs, corresponding to the higher prevalence of comorbidities in this group.

**Table 4:** **Antidiabetic and antihypertensive therapy by HbA1c and SBP targets at the six-month follow-up**

**Antidiabetic therapy**	**HbA1c ≤ 6.5%**		**HbA1c > 6.5% to ≤ 7.0%**		**HbA1c >7.0% to ≤ 7.5%**		p-value at 6 mo*
	6 mo	∆BL	6 mo	∆BL	6 mo	∆BL	
Metformin (%)	78.1	-0.6	80.9	-0.6	80.3	+0.9	<0.05
Sulfonylureas (%)	15.8	+0.6	19.1	-0.3	21.7	+0.1	<0.0001
Alpha-glucosidase inhibitors (%)	1.0	0.0	1.6	+0.1	0.8	-0.2	<0.05
DPP-4 inhibitors (%)	58.4	-0.6	66.7	+0.3	61.1	-2.2	<0.0001
Glinide (%)	2.5	+0.2	3.7	-0.1	6.6	+0.4	<0.0001
Short-acting insulin (%)	4.6	+0.9	5.3	+1.3	8.7	+2.0	<0.0001
Long-acting insulin (%)	10.3	+1.8	17.4	+2.7	21.7	+4.1	<0.0001
Mixed insulin (%)	0.9	+0.2	2.2	+0.3	3.7	+0.8	<0.0001
≥2 drugs (%)	60.9	+0.3	74.9	+1.5	78.2	+0.8	<0.0001
**Antihypertensive therapy**	**SBP ≤ 130 mmHg**		**SBP > 130 to ≤ 135 mmHg**		**SBP > 135 to ≤ 140 mmHg**		p-value at 6 mo*
	6 mo	∆BL	6 mo	∆BL	6 mo	∆BL	
ACE inhibitors (%)	50.9	-0.4	53.2	-0.8	54.2	-0.1	0.08
ARBs (%)	28.1	+0.4	28.9	+0.5	27.8	+0.5	0.73
Aliskiren (%)	0.4	-0.1	0.5	0.0	0.3	-0.1	0.74
Betablockers (%)	48.4	+0.6	47.8	+0.6	47.6	+0.7	0.87
Calcium blockers (%)	25.7	+0.6	29.3	+0.8	31.8	+1.0	<0.0001
Diuretics (%)	42.7	+0.4	42.9	+0.2	47.8	+1.3	<0.01
Other (%)	8.9	+0.2	10.5	+0.4	12.8	+0.1	<0.001
≥2 drugs (%)	63.3	+0.6	68.4	+0.3	70.0	+1.5	<0.0001

Legend: HbA1c, glycated hemoglobin; SBP, systolic blood pressure; *p-values compare the HbA1c and SBP groups, respectively.

### Six-month follow-up by HbA1c or SBP target group

**Figure 3:**
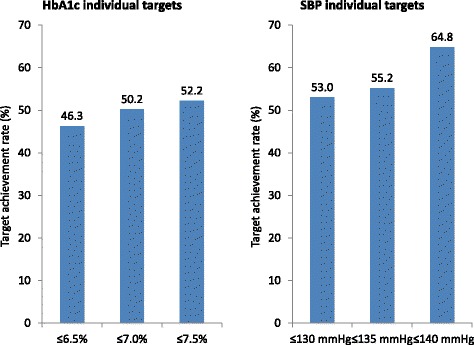
Target achievement rates at 6 months in each HbA1c and blood pressure target group.

At the 6-month follow-up (n = 6,586), we found that patients within the strict HbA1c treatment group had made more contacts with general practitioners (p < 0.0001, Table 5). Although patients within each target group showed reductions in mean glucose measurements, with postprandial glucose dropping to below 10 mmol/l in each group, overall HbA1c treatment goals remained unmet in all groups (Figure 3) with 46.3% achieving their pre-defined treatment target of ≤6.5% (50.2% >6.5 to ≤ 7.0%; 52.2% >7.0 to ≤7.5%). While the lowest mean HbA1c was achieved with strict treatment, the largest change in HbA1c from baseline was observed in the loose target group (p < 0.0001). In addition, when examining BP by HbA1c target group at follow-up, we observed reductions from baseline in both SBP and diastolic BP (DBP); however, there was not a significant difference between the groups with regard to the observed changes in SBP.

**Table 5:** **Patient data at six-month follow-up by HbA1c and SBP target group**

**HbA1c**	**HbA1c ≤ 6.5%(n = 2,647)**	**HbA1c > 6.5% to ≤ 7.0%(n = 2,790)**	**HbA1c >7.0% to ≤ 7.5%(n = 1,149)**	**P-values for the comparison of 3 groups**
GP contacts (number)	5.5 ± 4.8	4.7 ± 3.9	5.3 ± 4.4	<0.001
Specialist contacts (number)	2.3 ± 1.9	2.3 ± 2.7	2.3 ± 2.1	<0.05
Fasting glucose (mmol/l)	7.0 ± 2.0	7.4 ± 1.9	8.4 ± 2.8	<0.0001
Postprandial glucose (mmol/l)	8.4 ± 2.5	8.9 ± 2.7	9.8 ± 3.1	<0.0001
HbA1c (%)	6.8 ± 0.9	7.2 ± 0.9	7.7 ± 1.2	<0.0001
∆HbA1c from baseline (%)	-0.4 ± 1.2	-0.7 ± 1.2	-0.9 ± 1.5	<0.0001
SBP (mmHg)	135.3 ± 14.1	135.8 ± 13.9	136.7 ± 14.1	<0.01
∆SBP from baseline (mmHg)	-4.2 ± 16.7	-5.1 ± 15.6	-4.7 ± 15.5	0.06
DBP (mmHg)	80.8 ± 8.8	80.3 ± 8.5	80.4 ± 8.5	0.27
**SBP**	**SBP ≤ 130 mmHg (n = 2,602)**	**SBP > 130 to ≤ 135 mmHg (n = 2,225)**	**SBP > 135 to ≤ 140 mmHg (n = 1,729)**	**P-values for the comparison of 3 groups**
GP contacts (number)	5.5 ± 4.8	4.7 ± 3.7	5.2 ± 4.5	<0.0001
Specialist contacts (number)	2.3 ± 2.0	2.3 ± 2.4	2.3 ± 2.6	0.34
Fasting glucose (mmol/l)	7.2 ± 2.2	7.4 ± 2.0	7.7 ± 2.3	<0.0001
Postprandial glucose (mmol/l)	8.8 ± 2.7	8.9 ± 2.3	8.9 ± 3.3	<0.01
HbA1c (%)	7.0 ± 1.1	7.2 ± 1.0	7.2 ± 1.1	<0.0001
∆HbA1c from baseline (%)	-0.5 ± 1.2	-0.7 ± 1.2	-0.7 ± 1.3	<0.0001
SBP (mmHg)	133.4 ± 13.4	135.7 ± 13.3	139.1 ± 14.9	<0.0001
∆SBP from baseline (mmHg)	-3.0 ± 16.5	-5.2 ± 15.2	-6.4 ± 16.3	<0.0001
DBP (mmHg)	79.9 ± 8.3	80.5 ± 8.2	81.4 ± 9.4	<0.0001

Legend: HbA1c, glycated hemoglobin; SBP, systolic blood pressure; GP, general practitioner; DBP, diastolic blood pressure; data are provided as mean ± standard deviation.

Analyzing the 6-month follow-up data by SBP target group revealed that patients with strict SBP treatment had more contacts with general practitioners than the other groups (p < 0.0001; Table 5). Moreover, patients within each of the SBP target groups showed reductions in mean glucose measurements and HbA1c levels from baseline, with the strict SBP target group displaying the lowest values (p < 0.0001). Accordingly, the strict group also showed the least change from baseline HbA1c levels (p < 0.0001). With regard to BP measurements, we observed reductions from baseline SBP and DBP in all SBP target groups. Nevertheless, SBP targets were not reached at six months in the strict (53.0%) and medium (55.2%) treatment groups by approximately half of the patients only (Figure 3). In contrast, the SBP target was met in the loose treatment group in nearly 2/3 (64.8%) of the patients, which also showed the largest decrease in SBP from baseline (p < 0.0001).

**Figure 4:**
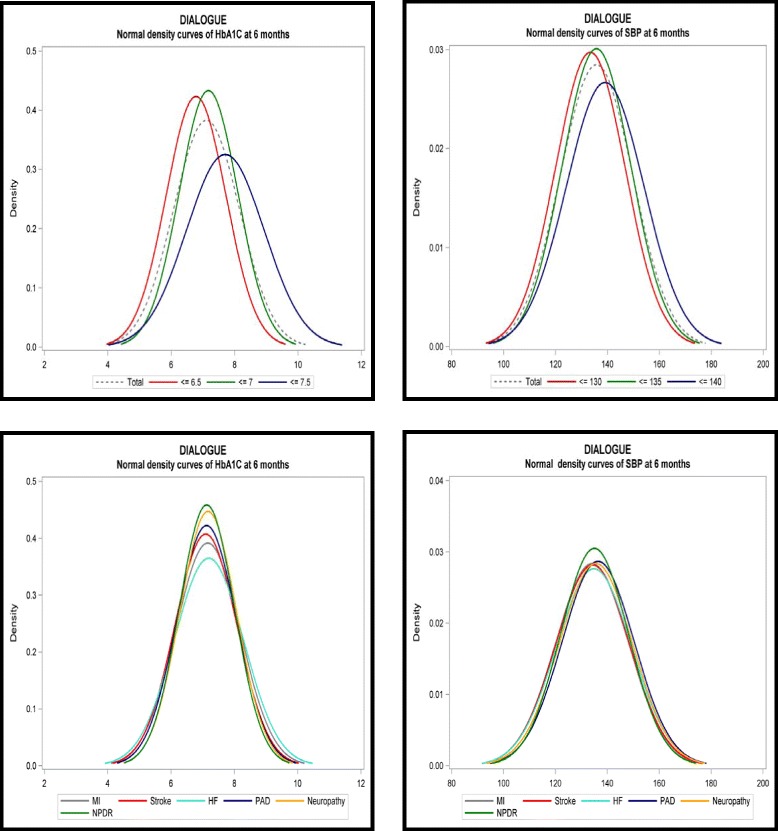
HbA1c/SBP distribution at 6 months overall and by comorbidity.

We also examined the overall distribution of 6-month outcomes for each of the distinct HbA1c target groups (Figure 4, upper panel). Interestingly, our analysis confirmed that the strictly targeted population as a whole maintained better glycemic control than the loose patient group. A similar, but less pronounced, result was observed when analyzing the different SBP treatment populations. In contrast, when considering the various patient populations based on specific comorbidities, we observed no difference in HbA1c or SBP treatment outcomes at six months (Figure 4, lower panel).

## Discussion

The DIALOGUE registry was designed to study the implementation and success of individualized treatment targets for patients with T2DM and hypertension in clinical practice. We found that patients with strict HbA1c or SBP targets were younger and displayed shorter disease duration, better glycemic control, lower BP, and less comorbid disease than those with less stringent treatment requirements. Thus, approximately 70% of patients were assigned to both strict HbA1c and SBP targeting by physicians. On the other hand, physicians opted for loose treatment goals in elderly individuals with more uncontrolled diabetes/BP and comorbidities (i.e., higher cardiovascular risk). Although individualized therapy led to substantial reductions in HbA1c and BP after 6-months of follow-up, treatment goals were often unmet and no further effort was made to improve the situation thereafter. Nevertheless, overall HbA1c and SBP levels remained lowest within the strictly treated patient population at the 6-month follow-up.

### Individualized treatment targets

Although individualized treatment goals have recently become a focus in the management of patients with T2DM [1], the efficacy and safety of such approaches must be carefully evaluated, and therapeutic criteria need to be better defined. Therefore, in addition to DIALOGUE, other recent studies have begun to evaluate the application and outcomes of individualized treatment guidelines in clinical practice. For example, the Diabetes in Germany (DIG) study compared various metabolic syndrome definitions and the consistency of guideline-oriented treatment across Germany [2-4], whereas, the DUTY registry evaluated whether treatment guidelines were effectively incorporated into the management of patients with diabetes in daily practice [5]. Overall, it was found that too many patients did not receive consistent therapy for cardiovascular risk factors according to guidelines, which meant that target values were rarely reached. Additionally, the prospective DiaRegis registry was designed to document the therapeutic course and outcomes of patients with T2DM in which initial antidiabetic therapies failed [6]. Among other results, it was found that that hypoglycemia was more frequent in T2DM patients with comorbid vascular disease [7]. Moreover, it has been reported that SBP remained uncontrolled in 50% of cases when using individualized treatment strategies for hypertension [7]. Our findings from the six-month DIALOGUE follow-up are in agreement with the notion that therapeutic targets are not currently being met with tailored treatments for diabetes and hypertension. Indeed, this is consistent with results from several recent reports that suggested suboptimal treatment target achievement with respect to both glucose and BP control [8-10]. The failure to achieve pre-defined treatment has been associated with inertia of physicians to make effort after an initial failure [11]. On the other hand multiple concomitant medications as well as treatment associated side-effects that become apparent after the defining the treatment target frequently require a reconsideration of these targets in an effort to balance benefits and risks of treatment [12]. Thus, it is essential that efforts be made not only to set appropriate treatment targets, but also to efficiently reach these goals in clinical practice. In this respect, it is important to determine the factors that play a role in the failure of current individualized treatment approaches in order to develop optimal strategies for improvement. Therefore, our findings from the DIALOGUE registry should contribute to the establishment and maintenance of specific individualized treatment targets for the management of patients with T2DM and hypertension.

### Trends in individualized treatment

Our findings revealed that patients who underwent strict treatment for T2DM and hypertension were younger and less comorbid than those with less stringent therapy goals. Interestingly, strictly managed patients also made more contacts with general practitioners and displayed lower rates of use and doses for various antidiabetic therapies. In this regard, the increased use of certain antidiabetic and antihypertensive treatments in patients with loose treatment goals may have contributed to the greater changes that we observed from baseline values for both HbA1c and SBP in this group. Indeed, the loose SBP target group was the only one to meet overall treatment goals in 2/3 of the patients. For reference the ESH/ESC Guidelines 2013 state that 70% is the target goal in populations [13]. It is interesting that these patients with less stringent treatment goals received heightened therapeutic intervention, as they were older and had more risk of complications and/or cardiovascular events. In this regard, there is a particular lack of data with regard to the use of pharmacologic agents in older individuals with T2DM, with clinical guidance largely based on data obtained from younger populations [14]. Thus, establishing and executing appropriate guidelines for individualized pharmacological therapy in older patients is of critical importance.

With regard to the standards used to assign patients for targeted therapy, our 6-month analysis of DIALOGUE revealed that a large proportion of patients were co-stratified into corresponding HbA1c and SBP target groups (i.e., 70% with strict HbA1c and strict SBP targeting). This may not be surprising considering that similar criteria might be used to establish individualized therapy for hypertensive or diabetic patients (e.g., comorbidities and cardiovascular risk) [15]. In this respect, we were able to determine specific predictors for choosing loose SBP or HbA1c treatment goals, including unique patient characteristics (e.g., SBP, FBG, HbA1c levels) as well as the presence of certain comorbidities (e.g., heart failure, PAD). Indeed, understanding how such factors are associated with specific treatment targets is essential for evaluating the clinical management of diabetic and hypertensive patients and determining which patients will respond well to tailored regimens. Thus, further study of these patient characteristics can ultimately ensure that therapeutic goals are met. However, since patients with both T2DM and hypertension require simultaneous targeting of BP and glucose levels [16], tailored therapies for these patients may also need to consider the effects of combined treatments.

### The role and impact of comorbidity

We found that comorbidity was a key factor that influenced the selection of individualized treatment in diabetic and hypertensive patients. In fact, those with comorbidities were less likely to receive strict treatment regimens in our registry. This is in line with the guidance of the American Diabetes Association on the management of hyperglycemia [17], suggesting the loosening of glycemic targets in patients with important comorbidities, established vascular disease and limited life expectancy.

Some studies have suggested that comorbidities in T2DM patients can actually prevent the achievement of good glycemic control [18-20], whereas others have argued against this association [21,22]. Nevertheless, Wilke et al. reported that T2DM patients reaching HbA1c goals along with additional treatment goals (i.e., for comorbidities) showed lower T2DM event rates, whereas subgroups failing to achieve one or several treatment goals presented greater risk [23]. That being said, multiple comorbidities can outbalance the benefit of tight glycemic control in elderly subjects due to the effects of hypoglycemia, thereby supporting looser treatment targets in older individuals [24,25]. In addition to the impact of hypoglycemia on comorbidities, comorbid conditions may also influence glycemic control. For example, the DiaRegis registry reported that hypoglycemia was more frequent in T2DM patients with vascular disease [26]. Indeed, it has been suggested that comorbid illnesses or functional impairments in older diabetic patients may constitute more important predictors of diminished benefit of intensive glucose control than age alone [27]. In contrast, the impact of comorbidities on achieving BP targets in high-risk patients is less well understood [28]. Stricter target BP is now recommended only in patients with chronic kidney disease or proteinuria and type 2 diabetes [13]. New studies will further provide important information on the risks and benefits of intensive BP treatment targets in patients with existing comorbidities (e.g., cardiovascular disease, chronic kidney disease) [29]. So far, in our 6-month analysis of DIALOGUE, we did not observe differential effects of specific comorbidities on overall treatment outcomes (i.e., no difference in six-month HbA1c or SBP outcomes when analyzing patient populations by specific comorbidities). Nevertheless, due to the potential impact that comorbidities have on both treatment strategies and patient outcomes, it was recently suggested that approaches allowing more complex comorbidity modeling might enhance the accuracy of individualized therapy [30]. Thus, more research on the epidemiology of complex comorbidities and how they interact with each other to change outcomes during tailored treatment of diabetic and/or hypertensive patients is warranted.

### Limitations

This investigation was subject to some limitations. Indeed, the patients analyzed in the present study were comorbid for T2DM and hypertension and were often administered combined therapy regimens which could have confounded data interpretation. However, since DIALOGUE is an observational study conducted in real clinical settings with physician-selected therapy, our results are highly representative of individualized treatment regimens currently employed for patients with T2DM and hypertension. In addition, it has been suggested that registry data can be less complete when compared to information collected in randomized clinical trials. However, three strategies were implemented to assure information quality, including front-end checks upon data entry, use of a sophisticated quality control program prior to creation of the analytic data set, and random site visits. Finally, it is possible that this 6-month follow-up was not sufficiently long enough to give an accurate picture of whether individualized BP and HbA1c treatment goals could be achieved in these patients.

## Conclusions

In patients with T2DM and hypertension, individualized glucose and BP targets are selected based on patient characteristics and overall comorbidity. Thus, those with low cardiovascular risk have stricter treatment goals. In this regard, patients with strict HbA1c targets also received strict treatment for SBP. Although individualized treatment led to substantial reductions in HbA1c and BP after 6-months of follow-up, for the most part, treatment goals were not achieved using various antidiabetic and antihypertensive therapies. However, as a whole, strictly treated patients maintained the lowest HbA1c and BP levels after 6 months of individualized treatment.
